# Parameter Sensitivity Analysis of Mounting Pedestals and Multi-Objective Optimization for a Multi-Support Rigid Body System

**DOI:** 10.3390/s22187067

**Published:** 2022-09-19

**Authors:** Qingyu Zhu, Qingkai Han, Xiaodong Yang, Junzhe Lin

**Affiliations:** 1 School of Mechanical Engineering, Dalian University of Technology, Dalian 116024, China; 2 School of Mechanical Engineering and Automation, Northeastern University, Shenyang 110819, China; 3 College of Mechanical Engineering, Beijing University of Technology, Beijing 100124, China

**Keywords:** multi-support rigid body system, structural optimization, sensitivity analysis, Sobol’s method, multi-objective optimization, NSGA-Ⅲ

## Abstract

In this paper, a parameter sensitivity analysis of mounting pedestals and a multi-objective optimization design for vibration reduction in a multi-support rigid body system, taking an aeroengine-lubricating oil tank supported by multiple mounting pedestals as an example, are conducted based on the third version of non-dominated sorting genetic algorithm (NSGA-Ⅲ) combined with Sobol’s sensitivity analysis method (SSAM). An aeroengine-lubricating oil tank with three mounting pedestals is simplified as a three-support dynamic system, and its dynamics model is established. Several structural parameters of mounting pedestals are taken as the design variables, and the system vibration response and the reaction force of the front and rear mounting pedestals are considered as the objective functions. The first-order results and total sensitivity index of different design parameters for each objective function are obtained via SSAM, and the five most sensitive parameters are selected. Based on the above five design parameters, multi-objective optimization designing for vibration reduction in a simplified lubricating oil tank system is conducted based on NSGA-Ⅲ, and the results of the above triple-objective optimization are obtained as a Pareto-front surface with an obvious frontier. It can be observed from the simulation results that the oil tank vibration of the optimized system is effectively suppressed under the unbalanced excitation of two typical engine speeds. The established method and the main results can provide guidance for designers of aeroengine external structural systems, which can help to achieve superior system dynamic performances in engineering applications.

## 1. Introduction

Some devices in industry engineering applications such as aerospace, energy and power, vehicle engineering, and the robotics field, are fixed by multi-point supports, and the composed systems have the typical characteristics of a rigid body system with spatial multi-point supports. Their dynamic characteristics are complex, and they are particularly prone to vibration, or even resonance, under external excitation [1,2,3]. For example, the lubricating oil tank of an aeroengine’s external device is fixed on the casing through spatial multi-point anchored supporting structures (usually three mounting pedestals or three supports with a tie rod and two mounting pedestals, and some are with four mounting pedestals as well) at different locations on a typical large-scale rigid body system with spatial multi-point supports. The lubricating oil tank system with multi-point supports has multiple excitations, a wide frequency domain, and a complex vibration response, and it is prone to strong vibration or even damage under aeroengine complex vibration excitations. Owing to the increasing demand for raising the thrust-to-weight ratio of modern aeroengines, higher requirements are placed on the strength, vibration and stability of aeroengine-lubricating oil tanks’ mounting pedestals. Therefore, in the design process of aeroengine-lubricating oil tanks and their mounting pedestals, it is of great need to carry out multi-objective optimization design for vibration reduction for a typical multi-support rigid body system.

Structural optimization is currently a frontier subject in engineering structural design, and it has a wide range of applications in engineering practice. It can be divided into size optimization, shape optimization, and topology optimization according to the type of design variables [4]. Due to the importance of structural optimization, the depth and breadth of research in this field have continued to expand, and many achievements have been obtained. Yang et al. [5] proposed a variety of stretcher structure optimization schemes based on the principle of dynamic vibration absorbers (DVAs). By optimizing the stretcher support stiffness and damping coefficient, the performance of the nonlinear vibration damping system of the tracked ambulance was improved, and vibration of the supine human body on the stretcher was reduced. The global sensitivity analysis method was used to calculate the sensitivity of the design parameters, and the non-dominated sorting genetic algorithm (NSGA-II) was used to optimize the nonlinear damping performance of the system. Caresta and Kessissoglou [6] used a resonant converter in the propulsion system to reduce the far-field radiated sound pressure from the submarine, and a combined genetic and pattern search algorithm was used to find the optimum virtual mass, stiffness, and damping parameters of the resonance converter. At present, most topology design studies are focused on single-objective topology optimization (SOTO), and some are also focused on multi-objective topology optimization (MOTO), but only focus on the effect of multi-stiffness on dynamic characteristics or vibration response [7,8]. Tan et al. [9] took stiffness and random vibration response as the optimization objectives, and applied multi-objective topology optimization to reduce the vibration of the primary supporting structure of the video satellite in the frequency domain. The dynamic analysis results showed that the root mean square values in all three spatial directions of the optimal structure by the multi-objective optimization were smaller than that of the single-objective optimization. The multi-objective optimization method significantly improved the vibration response of the base plate, which effectively suppressed the vibration of the satellite. Kapania et al. [10] summarized research on the structural optimization of stiffened plates, and pointed out that the optimization of the layout, thickness and height of the stiffeners was indispensable in practical applications. He et al. [11] adopt a data-driven method to study the multi-objective structural optimization problem, taking the layout, thickness, and height of the stiffeners as design variables to optimize the weight and mean square speed of stiffened plates. The numerical results showed that the proposed method was effective for the optimal design of stiffened plates and satisfied different band gap requirements in engineering. It can be observed that previous research on structural dynamic characteristics optimization was mostly focused on structure stiffness and damping parameters, ignoring structure geometry and mechanical parameters.

For sensitivity analysis (SA), a sensitivity analysis method that is very popular in many fields is the variance-based Sobol method. In general, variance-based sensitivity analysis methods aim to quantify the amount of variance that each parameter contributes to the unconditional variance of the model output [12,13]. Li et al. [14] proposed a novel sparse grid numerical integration method to estimate Sobol’s main effect indices, as well as the two decomposed sensitivity measures, and applied the proposed method to a roof truss and a composite beam model to understand the impact of correlation between variables on the output variance explicitly, greatly alleviating the burden of the curse of dimensions on high-dimensional problems. Wu et al. [15] took the uncertain variables of link lengths and joint rotation angles into account, and used Sobol’s method to analyze the sensitivity of uncertain variables for the positioning accuracy of industrial robots, by which the sensitive variables were determined to perform the positioning accuracy reliability analysis for industrial robots. Taking the bulb hydro generating unit (BHGU) as the research object, a nonlinear dynamic model of the BHGU with a shaft crack fault was established by Yan et al. [16]. The parameter sensitivity of the BHGU with the shaft crack fault was analyzed via Sobol’s method, and ranks of parameter sensitivity for the vibration displacement and unbalanced forces were obtained, respectively.

For optimization algorithm, the non-dominated sorting genetic algorithm (NSGA-II) was proposed by Deb [17] as an effective multi-objective evolutionary algorithm, and has been widely adopted by many researchers. Liu and Jiao [18] presented a multi-objective pipe-routing algorithm considering the vibration performance of an aeroengine based on the Kriging model and NSGA-II, where pipe length, smoothness and natural frequency were taken into account. The proposed algorithm can obtain a set of non-dominant pipeline layout solutions; especially, the natural frequency of the pipeline was far away from the engine excitation frequency, avoiding possible resonance. Chen et al. [19] defined the RMS of weighted acceleration, wheel dynamic load, and suspension dynamic deflection as evaluation indices of vehicle ride comfort, and established a multi-body dynamic rigid–flexible coupling model of an in-wheel motor vehicle. The relationship between vehicle ride comfort evaluation indices and suspension parameters was described by the Kriging model, and a multi-objective optimization using NSGA-II was processed using the above model, which improved the ride comfort performance. Zamanifar et al. [20] introduced an optimization technique based on NSGA-II, incorporating DFT analysis to achieve better control the performance of DFIG system stability. The NSGA-II was introduced and applied to solve the optimization problem of the propeller for high-altitude airships by Jiao et al. [21]. Aiming to design an efficient and lightweight propeller for high-altitude airships, the optimization model was established based on the conditions of high-altitude airship propulsion systems, and the effects of various design variables including pitch angle, chord length, diameter, rotational speed, and the number of blades on high-altitude propeller performance were presented and discussed. The third generation of the NSGA-III algorithm has been used for several years, and its performance is better than that of the second-generation algorithm. Based on human electroencephalogram (EEG) signals, Yi et al. [22] tested the performance of three crossover operators of the NSGA-III algorithm to solve a large-scale optimization problem The performance of the proposed NSGA-III variants was verified on six large-scale optimization problems. Aiming for a high-speed automotive turbocharger, Tarlani et al. [23] optimized the dynamic characteristics of the bearings, including the stiffness and damping coefficients, using the NSGA-III multi-objective optimization method to minimize vibrations caused by rotating unbalance. The results demonstrate that using optimized bearings can significantly improve the dynamic behavior of the rotor-bearing system, both at steady and transient operating conditions.

To summarize, the existing research on parameter sensitivity analysis and structure multi-objective topology optimization are separated. Sensitivity analysis can obtain ranks of parameter sensitivity, while subsequent optimization design is a direct step to solve engineering problems. Multi-objective topology optimization can obtain several design variables, while sensitivity analysis is the key step to select the most sensitive design variables. To the best of the author’s knowledge, Sobol’s sensitivity analysis method combined with the NSGA-III multi-objective optimization design for vibration reduction in an aeroengine-lubricating oil tank has never been reported. In this paper, an aeroengine-lubricating oil tank supported by multiple mounting pedestals is firstly simplified as a typical three-support system, and its dynamics model is established. Then, several structural parameters of mounting pedestals are taken as design variables, and the lubricating oil tank vibration response as well as the reaction force of the front and rear mounting pedestals are considered as the objective functions. Furthermore, the first-order results and total sensitivity index of different design parameters for each objective function are obtained, and the five most sensitive parameters are selected. Based on above five design parameters, multi-objective optimization design for vibration reduction in a simplified lubricating oil tank system is conducted based on NSGA-III, and the results of the triple-objective optimization are obtained with the Pareto-front surface with an obvious frontier. The simulation results show that the oil tank vibration of the optimized system is suppressed effectively under the unbalanced excitation of two typical engine speeds. The established method and main results can provide guidance for designers of aeroengine external structural systems, which can help to achieve superior system dynamic performances in engineering applications.

## 2. Dynamics Model of a Rigid Body System with Multi-Point Supports

**Figure 1:**
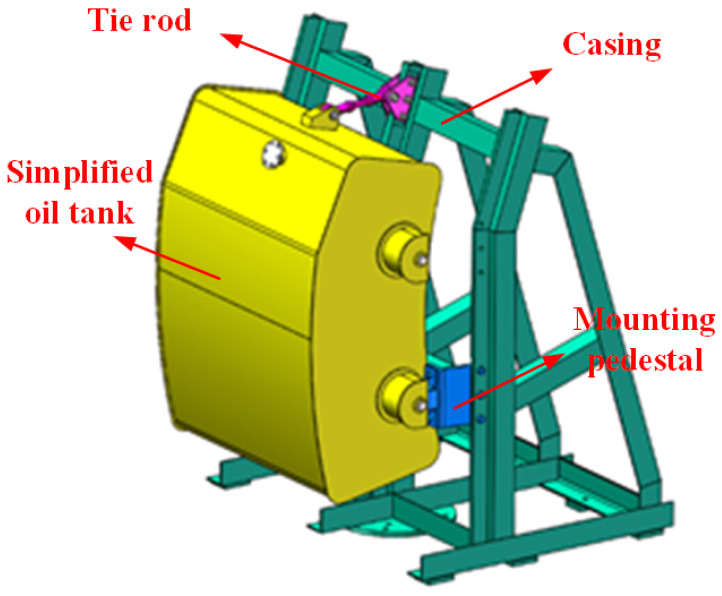
Simplified lubricating oil tank model supported by multiple mounting pedestals.

The specific structural object of an aeroengine-lubricating oil tank supported by multiple mounting pedestals can be simplified as a rigid body system with multi-point supports. The lubricating oil-tank is a typical three-support rigid body system that is mainly composed of an oil tank body, a casing, two fixed mounting pedestals, and an articulated mounting pedestal (tie rod), as shown in Figure 1.

The mounting location, mounting angle, elastic modulus of the mounting pedestals, cross-sectional area, initial length of the tie rod, and the damper damping coefficient of the mounting pedestals have a certain influence on the system’s vibration response and the reaction force of the mounting pedestals. A system dynamics model is established considering the above-mentioned mounting pedestal design parameters.

**Figure 2:**
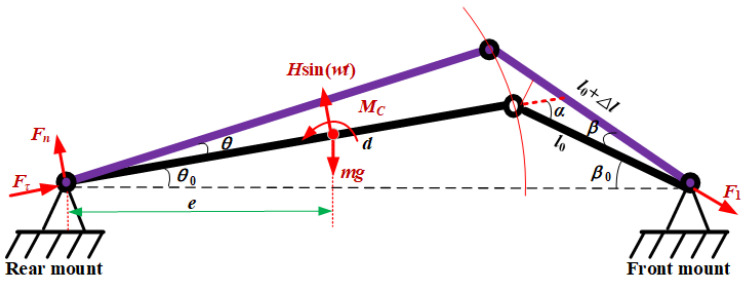
The simplified mechanical model of the oil tank supported by multiple mounting pedestals.

In order to the reduce vibration of the oil tank with a thin-walled structure, the internal supporting structure of the oil tank is mainly composed of a truss welded by a variety of square steel tubes. In the present study, the influence of the structural parameters of the mounting pedestals on the oil tank body vibration response is identified without considering the complex vibration of oil tank’s thin-walled structure, so the concentrated mass simplification of the oil tank is processed. Considering that the oil tank adopts a three-support type of tie rod and two mounting pedestals, the main vibration behavior of the oil tank is the rotation along the two fixed mounting pedestals, and herein the oil tank is simplified as a rigid rod and the tie rod is simplified as a flexible rod. The simplified mechanical model of the oil tank supported by multiple mounting pedestals is illustrated in Figure 2. In the above model, the initial length of the tie rod, the location of the rear mounting pedestals, and the distance from the center of mass to the rear support are denoted by *l*_0_, *d*, and *e*, respectively. *θ*_0_ and *β*_0_ denote the mounting angles of the mounting pedestals at two ends. The oil tank rotation angle is *θ*. The oil tank is subjected to the torque of the tie rod *M_C_*, the external excitation of the casing *H*sin(*ωt*), and its own gravity *mg*. The pulling force of the front mount by the tie rod is *F*_1_, and the reaction force of the rear mounting pedestals is *F*_2_; these are decomposed into *F*_n_ and *F*_τ_ along the normal and tangential directions of the simplified oil tank, respectively.

A force analysis of the simplified oil tank system is carried out and the forces are, respectively, decomposed along the normal and tangential directions of the oil tank’s rotational motion, obtaining

(1a) $$\text{Normal direction: }F_{t}+F_{1}\cos\alpha -mg\sin\theta_{0}=me{\left(\dot{\theta }\right)}^{2}$$

(1b) $$\text{Tan gential direction: }F_{n}+H\sin(\omega t)-F_{1}\sin\alpha -mg\cos\theta_{0}=me\ddot{\theta }$$

The reaction force of the rear mounting pedestals can be calculated as

(2) $$F_{2}=\sqrt{F_{t}{}^{2}+F_{n}{}^{2}}$$

Considering that the vibration response of the oil tank *θ* is small, the deformation of the tie rod can be expressed as

(3) $$\Delta l=d\theta \sin(\theta_{0}+\beta_{0})$$

Introducing the mounting angles of the oil tank $\alpha =\theta_{0}+\beta_{0}$, the tie rod tension can be obtained as

(4) $$F_{1}=\frac{EA}{l_{0}}\Delta l=\frac{EA}{l_{0}}d\theta \sin\alpha$$

where *E* represents the elastic modulus of tie rod and *A* represents the cross-sectional area of the tie rod.

The moment of the oil tank applied by the tie rod can be calculated as

(5) $$M_{c}=dF_{1}\sin\alpha =\frac{EA}{l_{0}}d\Delta l\sin\alpha$$

The oil tank torque balance equation can be described as

(6) $$I\ddot{\theta }+\gamma \dot{\theta }+M_{C}=0$$

where I represents the moment of inertia of the oil tank rotating along the mounting pedestals and γ represents the damping coefficient of the mounting pedestals.

Substituting Formulas (3) and (5) into Formula (6) yields

(7) $$I\ddot{\theta }+\gamma \dot{\theta }+\frac{EAd^{2}\sin^{2}\alpha }{l_{0}}\theta =0$$

The oil tank vibration differential equation can be written as

(8) $$\ddot{\theta }+\frac{\gamma }{I}\dot{\theta }+\omega_{n}{}^{2}\theta =0$$

The natural frequency of oil tank rotation is obtained as

(9) $$\omega_{n}{}^{2}=\frac{EA}{Il_{0}}{\left(d\sin\alpha \right)}^{2}$$

Assuming that the oil tank is subjected to external excitation Hsin(ωt) from the casing, etc., and the torque amplitude can be calculated as

(10) $$H=mae\omega^{2}$$

where *m* represents mass of the empty oil tank, *a* represents external acceleration excitation, and *e* is the distance from the center of mass of the oil tank to the rear mounting pedestals.

The forced vibration differential equation of the simplified oil tank system can be expressed as

(11) $$\ddot{\theta }+\frac{\gamma }{I}\dot{\theta }+\omega_{n}{}^{2}\theta =\frac{H}{I}\sin(\omega t)$$

Supposing that the equation is solved as

(12) θ=Xsin(ωt+φ)

Substituting Equation (12) into Equation (11) yields

(13a) $$X=\frac{H}{I\sqrt{{\left(\frac{\gamma \omega }{I}\right)}^{2}+{\left(\omega_{n}{}^{2}-\omega^{2}\right)}^{2}}}$$

(13b) $$\varphi =\arctan\frac{\gamma \omega }{I(\omega_{n}{}^{2}-\omega^{2})}$$

The vibration response of the oil tank can be obtained as

(14) $$\theta =\frac{H}{I\sqrt{{\left(\frac{\gamma \omega }{I}\right)}^{2}+{\left(\omega_{n}{}^{2}-\omega^{2}\right)}^{2}}}\sin(\omega t+\arctan\frac{\gamma \omega }{I{\left(\omega_{n}{}^{2}-\omega^{2}\right)}^{2}})$$

## 3. Design Parameters and Objective Functions of a Rigid Body System with Multi-Point Supports

In the design process of the oil tank and its mounting pedestals, the system vibration response amplitude |*θ*|, the front mount reaction force amplitude |*F*_1_| and the rear mount reaction force amplitude |*F*_2_| are defined as three objective functions. The mounting location of rear mounting pedestals *d*, mounting angle α, the elastic modulus *E*, cross-sectional area *A*, initial length *l*_0_ of the tie rod, the damping coefficient of the mounting pedestals γ, and the external acceleration excitation α are defined as decision variables. Sobol’s sensitivity analysis method (SSAM) is used to investigate the design parameters’ sensitivity to each objective function. Based on the above results, the NSGA-III is employed to optimize system objective functions.

### 3.1. Objective Functions

The three objective functions of the simplified oil tank system are defined as

(15) $$f_{1}(X)\overset{def}{\to }f_{1}(d,\alpha ,E,A,l_{0},\gamma ,a)=|\theta |=\frac{mae\omega^{2}}{I\sqrt{{\left(\frac{\gamma \omega }{I}\right)}^{2}+{\left(\omega_{n}{}^{2}-\omega^{2}\right)}^{2}}}$$

(16) $$f_{2}(X)\overset{def}{\to }f_{2}(d,\alpha ,E,A,l_{0},\gamma ,a)=|F_{1}|=|\frac{EA}{l_{0}}d\theta \sin\alpha |$$

(17) $$f_{3}(X)\overset{def}{\to }f_{3}(d,\alpha ,E,A,l_{0},\gamma ,a)=|F_{2}|=\sqrt{F_{\tau }{}^{2}+F_{n}{}^{2}}$$

### 3.2. Domain Intervals of Design Parameters

The design parameters of mounting pedestals are given in Table 1. The structural parameters, material parameters, and load parameters of mounting pedestals have a sufficiently wide range of values, which helps to obtain optimal results.

## 4. Sensitivity Analysis of Structural Parameters of Mounting Pedestals

In this section, the mounting location of rear mounting pedestals *d*, the mounting angle α, the elastic modulus *E*, cross-sectional area *A*, initial length *l*_0_ of the tie rod, the damping coefficient of the mounting pedestals γ, and the external acceleration excitation *a* are defined as decision variables, and SSAM is employed to investigate the design parameters’ sensitivity to each objective function.

### 4.1. Basic Principle of Sobol’s Sensitivity Analysis

The SSAM can not only quantify the influence degree of a single system parameter on the system, but can also analyze the influence degree of the simultaneous change of multiple parameters and the coupling effect between parameters on the system [12,13]. The basic process of SSAM is: (1) construct a mathematical model, which is the basis for the calculation of the sensitivity index; (2) clarify the domain interval of each design parameter; (3) sample the domain space of the design parameters and generate the corresponding sampling matrix; (4) calculate the parameter sensitivity index.

The mathematical model and parameter domain intervals are given in Section 3.1 and Section 3.2, respectively. The next step is to sample in the domain space of design parameters and generate the corresponding sampling matrix. Domain intervals of seven structural parameters are given, and we need to perform quasi Monte Carlo sampling on the design parameters via the Sobol method to generate two mutually independent *m×n* sampling matrices, *A* and *B*, where *m* is the number of samples for each parameter and the number is taken as *m* = N, n is the number of design parameters (*n* = 7), and the dimensions of the sampling matrices *A* and *B* are N×7. Meanwhile, another two matrices $A_{B}^{\left(i\right)}$ and $B_{A}^{\left(i\right)}$, are defined on the basis of the sampling matrices *A* and *B*. $A_{B}^{\left(i\right)}$ denotes that the matrix *A* is taken as the main body, and the *i*-th column element of matrix *A* is replaced by the *i*-th column element of matrix *B*, and the other six column elements in matrix *A* remain unchanged. The matrix $B_{A}^{\left(i\right)}$ can be obtained in the same way. Finally, approximate calculations of *V_i_* and *V_Ti_* are performed, according to different combinations of matrices *A*, *B*, and $A_{B}^{\left(i\right)}$, $B_{A}^{\left(i\right)}$.

(18) $$E(Y)=\frac{1}{N}\sum_{m=1}^{N}f{\left(A\right)}_{m}$$

(19) $$V_{i}=\frac{1}{N}\sum_{m=1}^{N}f{\left(B\right)}_{m}(f{\left(A_{B}{}^{\left(i\right)}\right)}_{m}-f{\left(A\right)}_{m})$$

(20) $$V_{Ti}=\frac{1}{2\times N}\sum_{m=1}^{N}(f{\left(A\right)}_{m}-f{\left(A_{B}{}^{\left(i\right)}\right)}_{m}{)}^{2}$$

(21) $$V=\frac{1}{N}\sum_{m=1}^{N}f{\left(A\right)}^{2}{}_{m}-{\left[\frac{1}{N}\sum_{m=1}^{N}f(A)\right]}^{2}$$

*m* represents the *m*-th row of the corresponding sampling matrix, and the row vector in the matrix can be expressed as

(22) $$x=(x_{1},x_{2},x_{3},x_{4},x_{5},x_{6},x_{7},x_{8})$$

where *x*_1_ represents the mounting location of the rear mounting pedestals *d*; *x*_2_ represents mounting angle α; *x*_3_, *x*_4_, and *x*_5_ represent the elastic modulus *E*, cross-sectional area *A*, and the initial length *l*_0_ of the tie rod, respectively; *x*_6_ represents the damping coefficient of the mounting pedestals γ; and *x*_7_ represents the external acceleration excitation *a*.

The estimation of the first-order influence index *S_i_* and total sensitivity index *ST_i_* can be expressed as

(23) $$S_{i}=\frac{V_{i}}{V}$$

(24) $$ST_{i}=\frac{V_{Ti}}{V}$$

The first-order influence index *S*_i_ reflects the contribution degree of a single design parameter *X*_i_ to the output result. The total sensitivity index *ST*_i_ reflects the influence of a single design parameter *X*_i_ on the output result and the influence of the interaction effect between *X*_i_ and the other parameters on the output result.

Finally, the above-mentioned calculation process is simulated via MATLAB software, and the influence weight of each structural parameter of the simplified lubricating oil tank system on each objective function can be obtained. Furthermore, the total sensitivity index of each parameter can be calculated.

### 4.2. Sensitivity Analysis Results

The more samples are taken, the more the calculation results tend to converge. Numbers of samples are taken as *m*= (7, 507, 1007, 1507, …, 9507), and the first-order influence index *S*_i_ and total sensitivity index *ST*_i_ of each parameter are calculated.

**Figure 3:**
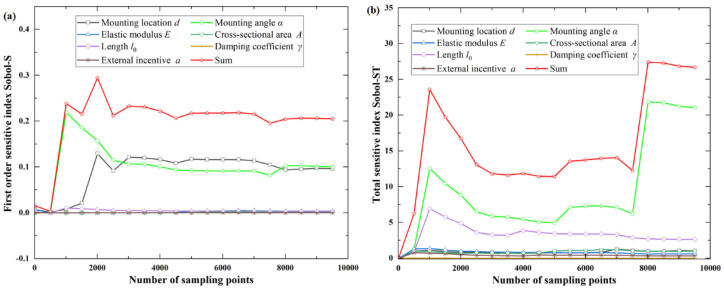
First-order and total sensitive index for objective function *f*_1_(***X***): (**a**) first-order sensitive index Sobol−S; (**b**) total sensitive index Sobol−ST.

For the objective function *f*_1_(***X***), the total sensitivity index of system parameters is much larger than the first-order sensitivity index of system parameters, indicating that the coupling effect of different parameters is significant. It can be observed from the total sensitivity index that the mounting angle α has the greatest influence on the function, followed by the initial length of the tie rod *l*_0_ and the mounting location of the oil tank *d*, as shown in Figure 3 and Table 2.

**Figure 4:**
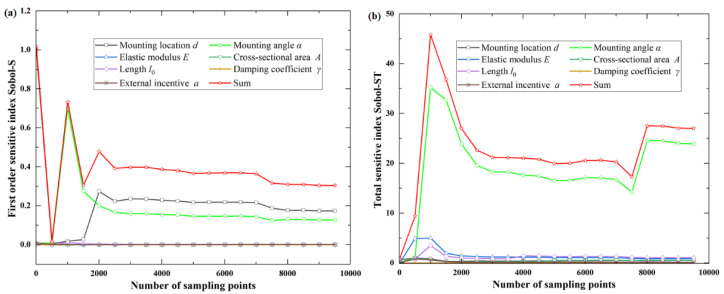
First-order and total sensitive index for objective function *f*_2_(***X***): (**a**) first-order sensitive index Sobol−S; (**b**) total sensitive index Sobol−ST.

For the objective function *f*_2_(***X***), it can be observed from the total sensitivity index that the mounting angle α has the greatest influence on the function, and the coupling effect of other design parameters is not obvious. It can be observed from the first-order sensitivity index that the mounting location *d*, the mounting angle α of the oil tank, and the initial length of the tie rod *l*_0_ are the three most sensitive parameters, in turn, as shown in Figure 4 and Table 2.

**Figure 5:**
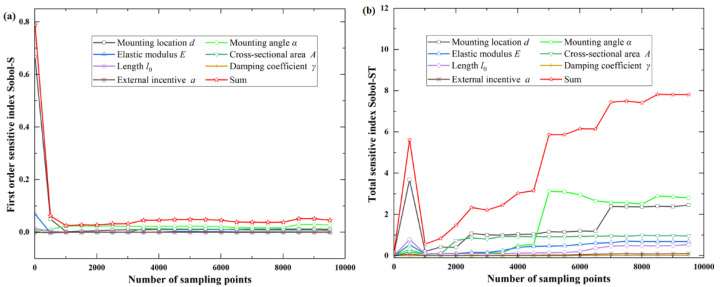
First-order and total sensitive index for objective function *f*_3_(***X***): (**a**) first-order sensitive index Sobol−S; (**b**) total sensitive index Sobol−ST.

For the objective function *f*_3_(***X***), the coupling effect between the various system parameters is significant. It can be observed from the total sensitivity index that the mounting angle α and mounting location *d* of the oil tank have the greatest influence on the function, and they are almost equal, followed by cross-sectional area *A* and the elastic modulus *E* of the tie rod, as shown in Figure 5 and Table 2.

Furthermore, the specific first-order sensitivity index and total sensitivity index of each parameter are calculated, as listed in Table 2.

## 5. Multi-Objective Optimization of a Rigid Body System with Multi-Point Supports

Based on the sensitivity analysis results in Section 4.2, the mounting location of the rear mounting pedestals *d*, the mounting angle α, the elastic modulus *E*, the cross-sectional area *A*, and the initial length *l*_0_ of the tie rod are defined as decision variables, and NSGA-III is employed to optimize the system’s objective functions.

### 5.1. Definition of Pareto Optimal Solution

Generally, the mathematical model of a multi-objective optimization problem can be expressed as

(25) $$\{\begin{array}{c} \min y=F(x)=(f_{1}(x),f_{2}(x),\ldots ,f_{m}(x))\\ s.t.g_{l}(x)\leq 0,i=1,2,\ldots ,q\\ h_{l}(x)=0,j=1,2,\ldots ,p \end{array}$$

where $x=(x_{1},\ldots ,x_{n})\in X\subset R^{n}$ is an n-dimensional decision vector, *X* is defined as an n-dimensional decision space, $y=(y_{1},\ldots ,y_{n})\in Y\subset R^{n}$ is an m-dimensional object vector, and *Y* is an m-dimensional object space. Additionally, $g_{l}(x)\leq 0,i=1,2,\ldots ,q$ represents *q* inequality constraints and $h_{l}(x)=0,j=1,2,\ldots ,p$ represents p equality constraints.

For multi-objective optimization problems there is usually a set of solutions, and it is impossible to improve any objective function without weakening at least one other objective function. Such solutions are called nondominated solutions, or Pareto optimal solutions, and are defined as follows:

**Definition** **1** **(feasible solution):** *for* x∈X *, if x satisfies the constraints in Formula (25), such as inequality* $g_{l}(x)\leq 0,i=1,2,\ldots ,q$ *and equation* $h_{l}(x)=0,j=1,2,\ldots ,p$ *, then x is called the feasible solution of the problem.*

**Definition** **2** **(feasible** **solution** **set):** *the set of all feasible solutions in X is called the feasible solution set, denoted by *$X_{f}$*, *$X_{f}\subseteq X$.

**Definition** **3** **(Pareto** **dominated):** *assuming* $x_{A},x_{B}\in X_{f}$ *are two feasible solutions, if*

(26) $$f_{l}(x_{A})\leq f_{l}(x_{B})\wedge \exists l=1,2,\cdots ,m,f_{l}(x_{A})<f_{l}(x_{B})\;$$

*then it is called *$x_{A}$* dominant *$x_{B}$*, denoted by *$x_{A}≺x_{B}$.

**Definition** **4** **(Pareto** **optimal** **solution):** *a solution,* $x^{*}\in X_{f}$ *, is called a non-dominated solution if and only if*

(27) $$\neg \exists \;x\in X_{f}\;:x≺x^{*}$$

**Definition** **5** **(Pareto** **optimal** **solution** **set):** *the Pareto optimal solution set is the set of all non-dominated solutions, which is defined as follows*

(28) $$P^{*}=\left\{x^{*}|\neg \exists \;x\in X_{f}\;:x≺x^{*}\right\}$$

**Definition** **6** **(Pareto-front** **surface):** *defining the Pareto optimal solution set as* $P^{*}$ *, the Pareto-front surface* $PF^{*}$ *can be defined as*

(29) $$PF^{*}=\left\{F(x^{*})=(f_{1}(x^{*}),f_{2}(x^{*}),\ldots ,f_{m}(x^{*}))\right\}$$

### 5.2. Steps of NSGA-III Algorithm

**Figure 6:**
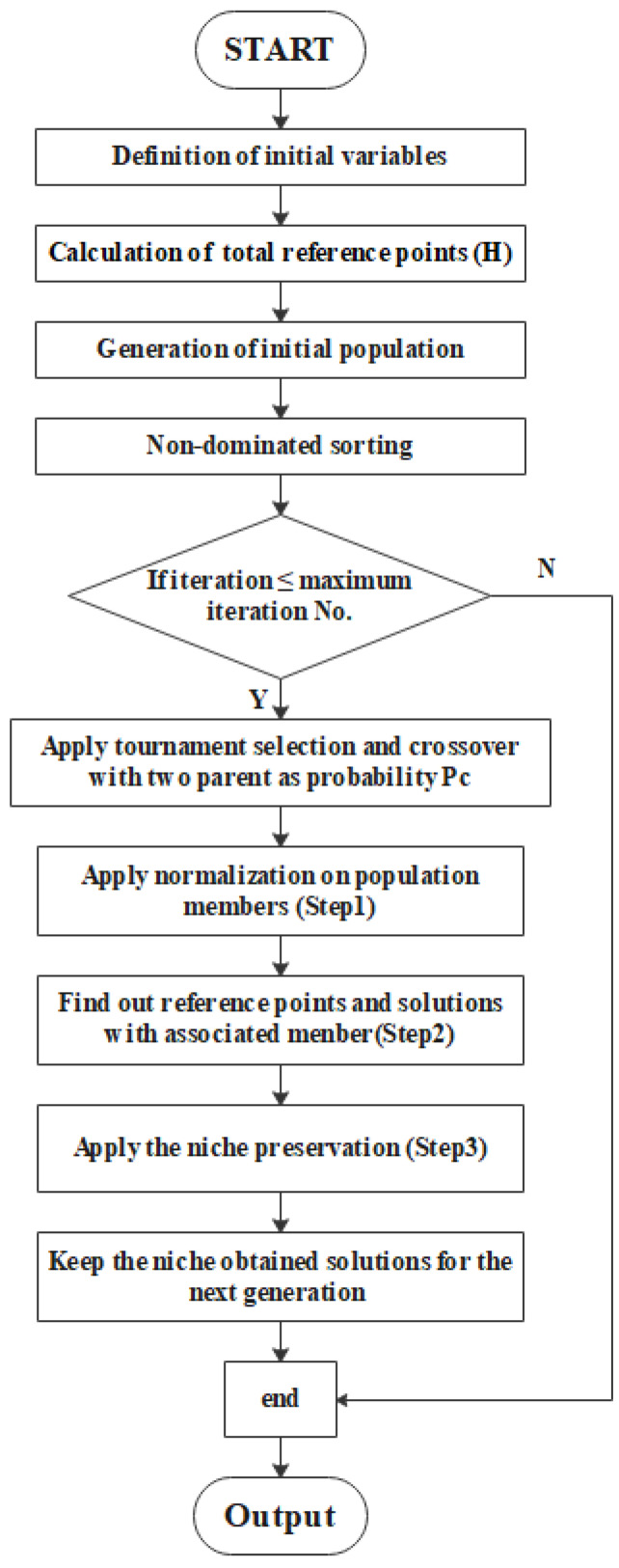
Algorithm flow of NSGA-III.

The traditional multi-objective optimization of evolutionary algorithms (MOEAs) cannot satisfy the requirements for online data processing because of its high computational costs. This drawback makes it difficult for MOEAs to solve multi-objective, large-scale optimization problems. Among different evolutionary algorithms, the NSGA-III is a fairly new method capable of solving large-scale optimization problems with a good ability to search for Pareto optimal solution sets. The main idea of NSGA-III is to introduce a reference point mechanism on the basis of NSGA-II and retain those individuals that are non-dominated and close to the reference points. NSGA-III and NSGA-II have similar frameworks. The main difference between the two algorithms is the selection mechanism. NSGA-II is mainly sorted by crowding degree, and its effect is not obvious in high-dimensional objective function spaces. NSGA-III makes a drastic adaptation of the crowding degree, maintaining population diversity by widely introducing distributed reference points. The flow chart of the proposed scheme is presented in Figure 6.

### 5.3. Optimization Results and Verification

Based on the proposed mathematic model, tri-objective optimization was carried out using the NSGA-II and NSGA-III algorithms. Then, vibration responses under the unbalanced excitation of two typical engine speeds (*n*_1_ = 10,200 r/min and *n*_2_ = 13,200 r/min) were calculated to validate the optimization results.

**Figure 7:**
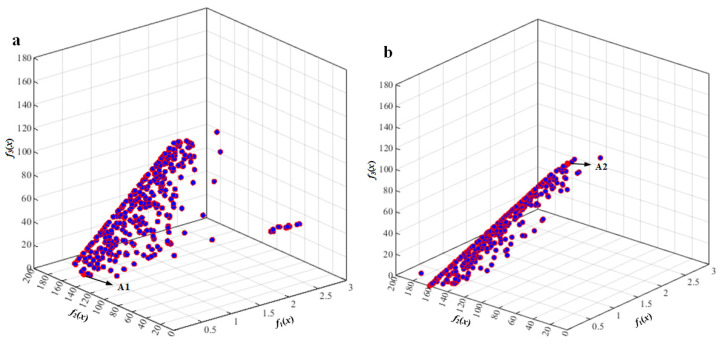
Pareto front obtained by tri-objective optimization using NSGA-III under engine rotation speed *ω*_1_ = 170 Hz: (**a**) the optimal solution A1; (**b**) the optimal solution A2.

**Figure 8:**
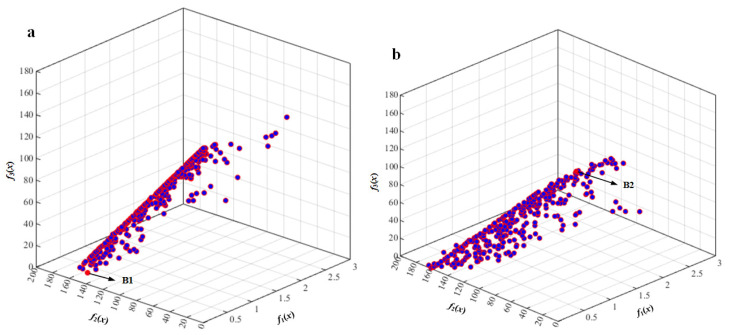
Pareto front obtained by tri-objective optimization using NSGA-III under engine rotation speed *ω*_2_ = 220 Hz: (**a**) the optimal solution B1; (**b**) the optimal solution B2.

Figure 7 is the Pareto front obtained by NSGA-III optimization with tri-objective functions under engine rotation speed *n*_1_ = 10,200 r/min (*ω*_1_ = 170 Hz). The results of points A1 and points A2 obtained by NSGA-II and NSGA-III optimization are listed in Table A1. It can be seen that A1 has the lowest vibration response and reaction force of the rear mount, while it has the largest reaction force of the front mount. A2 has the lowest vibration response and reaction force of the front mount, while it has the largest reaction force of the rear mount. The optimized results are similar under the unbalanced excitation of engine speed *n*_2_ = 13,200 r/min (*ω*_2_ = 220 Hz), as shown in Figure 8.

In order to validate the optimization results and compare the two optimization methods, a reference point P_ref.A_ with mounting pedestal parameters given in Table A1 is selected, and its corresponding objective functions under the unbalanced excitation of engine speed *ω*_1_ = 170 Hz were calculated. It can be observed from the optimization results in Table A1 that the vibration of point A1 obtained via NSGA-II dropped 35% and the reaction force of the rear mount dropped 80%, compared with those of point P_ref.A_. Additionally, the vibration of point A1 obtained via NSGA-III dropped 44% and the reaction force of the rear mount dropped 94%, compared with those of point P_ref.A_. The vibration of point A2 dropped 41% and the reaction force of the front mount dropped 94%, compared with those of point P_ref.A_. Additionally, the vibration of point A2 obtained via NSGA-III dropped 59% and the reaction force of the rear mount dropped 91%, compared with those of point P_ref.A_. The optimization results obtained via NSGA-III were better than those of NSGA-II. When the simplified lubricating oil tank was subjected to the unbalanced excitation of engine speed *ω*_2_ = 220 Hz, the optimization results were similar to those of the unbalanced excitation of engine speed *ω*_1_ = 170 Hz.

**Figure 9:**
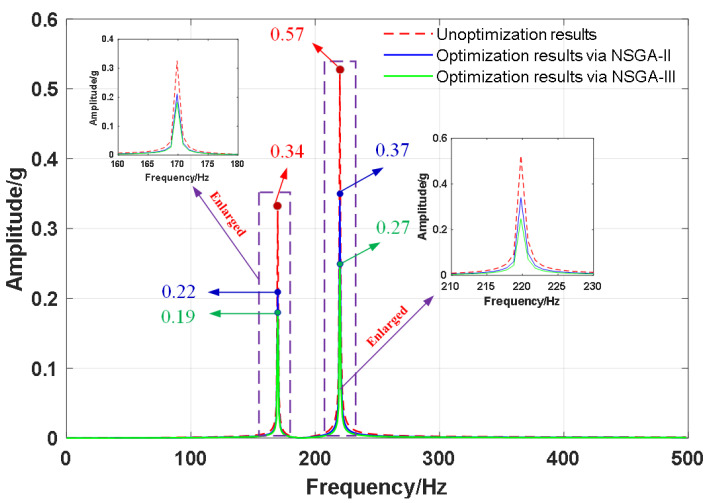
Vibration response of optimization results.

The vibration response of the two optimized results (points A1 and Pref.A, points B1 and P_ref.B_) under different unbalanced excitation engine speeds are shown in Figure 9. It can be seen that as the speed increases, the vibration response of the oil tank increases. Compared with the vibration response of the unoptimized points (P_ref.A_ and P_ref.B_), that of the optimized points (points A1 and points B1) drops significantly, and the optimization results obtained via NSGA-III are better than those of NSGA-II.

## 6. Conclusions

In the current study, the non-dominated sorting genetic algorithm (NSGA-III) combined with Sobol’s sensitivity analysis method has been employed to optimize an aeroengine simplified lubricating oil tank supported by multiple mounting pedestals. In the optimization process, several structural parameters of mounting pedestals are taken as the design variables, and the system vibration response as well as the reaction forces of the front and rear mounting pedestals are considered as the objective functions. The results of the triple-objective optimization are obtained as Pareto-front surfaces. The following conclusions are obtained:

- By Sobol’s sensitivity analysis method, the importance ranking of mounting pedestals parameters can be obtained. Many other sensitivity analysis problems in engineering applications can adopt the method used in this paper.
- By using NSGA-III, multi-objective optimization problems of vibration reduction for aeroengines with simplified lubricating oil tanks supported by multiple mounting pedestals can be solved efficiently. The Pareto optimal solution set can be obtained, by which the designer can consider different objective functions simultaneously and make final decisions according to design demands. The optimization results are verified, and the system vibration is suppressed effectively under the unbalanced excitation of two typical engine speeds (*n*_1_ = 10,200 r/min and *n*_2_ = 13,200 r/min). Many other multi-objective optimization problems in engineering applications can adopt the method used in this paper.
- The optimization results of mounting pedestal parameter design for vibration reduction in a multi-support rigid body system can help to achieve better system dynamic performances according to design requirements in engineering applications. They can contribute to the practical application of structural optimization in engineering applications in order to reduce vibration and reaction force level, and improve the service time of key parts, such as mounting pedestals, in aeroengine-lubricating oil tanks.

## Figures and Tables

**Table 1:** Domain intervals of design parameters.

Design Parameters	*d*	α (°)	*E* (Pa)	*A* (m^2^)	*l*_0_ (m)	*γ*	*a* (g)
*Variables*	*x*(1)	*x*(2)	*x*(3)	*x*(4)	*x*(5)	*x*(6)	*x*(7)
*Max*	1	4π/6	1 × 10^12^	1 × 10^−3^	0.15	1	50
*Min*	0	0	1 × 10^10^	1 × 10^−5^	0.05	1 × 10^−3^	1

**Table 2:** First-order and total sensitive index of each parameter for different objective functions.

		*d*	*α*	*E*	*A*	*l*_0_	*γ*	*a*
*f_1_(**X**)*	**S**	0.0961	0.1003	0.0037	0.0017	0.0029	0.0001	0.0001
	**ST**	1.0511	21.058	0.6180	0.9934	2.6356	0.0013	0.3299
*f_2_(**X**)*	**S**	0.1732	0.1272	0.0005	−0.0001	0.0018	−0.0001	0.0014
	**ST**	0.5209	23.908	0.8830	0.5342	1.0690	0.0004	0.1209
*f_3_(**X**)*	**S**	0.0112	0.0274	0.0006	0.0064	−0.0010	0.0001	0.0008
	**ST**	2.4555	2.8011	0.6885	0.9611	0.5323	0.0145	0.0970

## Data Availability

Not applicable.

## Appendix A

**Table A1:** Comparison of optimization results.

			*d*	*α* (°)	*E* (Pa)	*A* (m^2^)	*l*_0_ (m)	*f*_1_(*X*) (g)	*f*_2_(*X*) (N)	*f*_3_(*X*) (N)
**170 Hz**		P_ref.A_	0.50	60	5 × 10^10^	1 × 10^−4^	0.06	0.34	84	127
	**NSGA-II**	A1	0.21	1.88	2.27 × 10^10^	1.25 × 10^−4^	0.08	0.22 (↓35%)		25 (↓80%)
		A2	0.21	1.24	1.08 × 10^10^	1.87 × 10^−4^	0.06	0.20 (↓41%)	5 (↓94%)	
	**NSGA-III**	A1	0.29	1.95	4.40 × 10^10^	1.09 × 10^−5^	0.12	0.19 (↓44%)		7 (↓94%)
		A2	0.30	2.00	1.00 × 10^10^	4.64 × 10^−5^	0.09	0.14 (↓59%)	7 (↓91%)	
**220 Hz**		P_ref.B_	0.50	60.00	5.00 × 10^10^	1.00 × 10^−4^	0.06	0.57	84	127
	**NSGA-II**	B1	0.14	1.94	1.87 × 10^10^	3.32 × 10^−4^	0.08	0.37 (↓33%)		56 (↓56%)
		B2	0.15	1.95	1.86 × 10^10^	3.19 × 10^−4^	0.08	0.38 (↓33%)	13 (↓85%)	
	**NSGA-III**	B1	0.12	1.98	4.04 × 10^11^	1.00 × 10^−5^	0.14	0.27 (↓53%)		4 (↓96%)
		B2	0.18	2.01	3.60 × 10^10^	4.00 × 10^−5^	0.10	0.27(↓53%)	8 (↓90%)	
